# Which factors predict tumor recurrence and survival after curative hepatectomy in hepatocellular carcinoma? Results from a European institution

**DOI:** 10.1186/s12893-024-02399-y

**Published:** 2024-04-08

**Authors:** Sascha Vaghiri, Dimitrios Prassas, Onur Mustafov, Sinan Kalmuk, Wolfram Trudo Knoefel, Nadja Lehwald-Tywuschik, Andrea Alexander, Levent Dizdar

**Affiliations:** 1 Department of Surgery (A), Heinrich-Heine-University and University Hospital Duesseldorf, Moorenstr. 5, Bldg. 12.46, 40225 Duesseldorf, Germany; 2 Department of Surgery, Katholisches Klinikum Essen, Philippusstift, Teaching Hospital of Duisburg-Essen University, Huelsmannstrasse 17, 45355 Essen, Germany

**Keywords:** Overall and disease free survival, Hepatocellular carcinoma, Predictive factors, Curative resection

## Abstract

**Background:**

High tumor recurrence and dismal survival rates after curative intended resection for hepatocellular carcinoma (HCC) are still concerning. The primary goal was to assess predictive factors associated with disease-free (DFS) and overall survival (OS) in a subset of patients with HCC undergoing hepatic resection (HR).

**Methods:**

Between 08/2004–7/2021, HR for HCC was performed in 188 patients at our institution. Data allocation was conducted from a prospectively maintained database. The prognostic impact of clinico-pathological factors on DFS and OS was assessed by using uni- and multivariate Cox regression analyses. Survival curves were generated with the Kaplan Meier method.

**Results:**

The postoperative 1-, 3- and 5- year overall DFS and OS rates were 77.9%, 49.7%, 41% and 72.7%, 54.7%, 38.8%, respectively. Tumor diameter ≥ 45 mm [HR 1.725; (95% CI 1.091–2.727); *p* = 0.020], intra-abdominal abscess [HR 3.812; (95% CI 1.859–7.815); *p* < 0.0001], and preoperative chronic alcohol abuse [HR 1.831; (95% CI 1.102–3.042); *p* = 0.020] were independently predictive for DFS while diabetes mellitus [HR 1.714; (95% CI 1.147–2.561); *p* = 0.009), M-Stage [HR 2.656; (95% CI 1.034–6.826); *p* = 0.042], V-Stage [HR 1.946; (95% CI 1.299–2.915); *p* = 0.001, Sepsis [HR 10.999; (95% CI 5.167–23.412); *p* < 0.0001], and ISGLS B/C [HR 2.008; (95% CI 1.273–3.168); *p* = 0.003] were significant determinants of OS.

**Conclusions:**

Despite high postoperative recurrence rates, an acceptable long-term survival in patients after curative HR could be achieved. The Identification of parameters related to OS and DFS improves patient-centered treatment and surveillance strategies.

## Background

HCC is the most frequently diagnosed primary hepatic malignancy and the third leading cause of cancer related death globally [1, 2]. The most relevant factor for HCC development is liver cirrhosis [2]. Other predisposing risk factors are viral, toxic, metabolic, and immuno-related [3]. Operative approaches including HR and liver transplantation (LT) are the most potential curative treatment options in patients with resectable HCC providing long term cancer free survival [4, 5]. LT is associated with a significant reduction of HCC recurrence compared to HR [6] as it not only removes the tumor but also the surrounding cirrhotic liver remnant and reduces the risk of *de-novo* HCC formation [7–10]. However, the shortage of liver allografts and the high drop-out rates during the waiting period precludes the widespread application of LT for HCC [4, 11, 12]. Continuing technical evolvements, improved perioperative management, and proper patient selection in recent years have resulted in significant decreasing morbidity and mortality rates across experienced western and eastern institutions following HR and expanded the pool of patients even with relevant co-morbidities as potential candidates for surgical resection [13–15]. Indeed, HR has been successfully applied in patients with advanced tumor stages demonstrating promising oncological results [16, 17].

However, long-term outcomes after liver resection of HCC remain discouraging as tumor recurrence after curative intended HR is still very high with reported recurrence rates of 62.5%-72.7% [18–21]. The complex and heterogeneous HCC nature, underlying hepatic disease conditions and different patient populations hamper precise tumor relapse prognosis [22]. Hence, it is crucial to identify predictive survival factors after hepatectomy and to integrate them into the clinical decision-making process as well as into preventive and postoperative screening strategies in order to estimate prognosis and improve long-term oncological outcomes [21, 23]. Recently, a new adjuvant immunotherapy with autologous cytokine-induced killer cells increased recurrence-free and overall survival in patients with high-risk of recurrence after curative HCC treatment compared to the control group [24].

Therefore, the primary aim of our study was to assess the relationship between tumor recurrence and survival with relevant clinico-pathological variables of mostly cirrhotic patients undergoing curative HR.

## Methods

### Patients and study design

All patients with first time curative intended HR due to HCC between August 2004 and July 2021 were identified within the prospectively maintained oncological database from the Department of General, Visceral and Pediatric Surgery at the Heinrich-Heine-University Duesseldorf, Germany and subsequently included in the final analysis. The exclusion criteria were as following: patient age < 18 years (*n* = 2), non-HCC pathology (*n* = 2), death within 30 postoperative days (*n* = 8), and lost to follow-up or incomplete follow-up data respectively (*n* = 4). This study was approved by the local ethics committee of the Heinrich-Heine-University Duesseldorf, Germany (study-no.: 2021–1800- KFogU) and was conducted in strict accordance with latest version of the Declaration of Helsinki and the “Strengthening the Reporting of Observational Studies in Epidemiology” (STROBE) checklist for observational Studies [25]. Informed consent was waived because no data regarding the cases were disclosed. The primary endpoint was to calculate disease-free survival (DFS) and overall survival (OS) rates and to identify predictive survival factors in patients after hepatectomy for HCC.

### Clinico-pathological parameters

The following data were extracted for each eligible patient with regard to documented pre-and postoperative, surgical and pathological information:

1.  Preoperative: demographics including age, gender, BMI (body mass index), ASA score (American Society of Anesthesiologists), relevant co-morbidities, pre-existing hepatic disease, laboratory findings (liver function tests, total blood count, renal parameters, albumin, α-fetoprotein, hepatitis serology), MELD Score (Model for End-Stage Liver Disease), Child–Pugh classification, preoperative imaging studies with number, size, and location of tumor nodules, volumetric liver calculation and/or hepatobiliary scintigraphy assessment (HBS) in case of insufficient or critical future liver remnant (FLR).
2. Intraoperative: type and extent of resection, biliary or vascular resection and reconstruction, duration of surgery (minutes), number of transfused blood units.
3. Postoperative: morbidity including bile leakage, intra-abdominal abscess formation, cholangitis, sepsis, wound infection, and liver failure classification according to the International Study Group of Liver Surgery (ISGLS)´criteria [26].
4. Pathology: total number of tumors, maximum tumor diameter (mm), TNM classification based on UICC (Union internationale contre le cancer) 8th edition [27], tumor grading, width of resection margin, lymphangio-invasion (L-Stage), lymph node status (N-Stage), and micro- or macro-vascular tumor infiltration (V-Stage).

Type and extent of hepatic resection were defined according to the current available terminology for hepatectomy [28]. Major hepatectomy was considered if ≥ 3 segments were removed. Postoperative morbidity was stratified based on the Clavien-Dindo classification [29]. Sepsis criteria were evaluated based on the latest international consensus guidelines (Sepsis-3) [30]. The definition of preoperative chronic alcohol abuse was based on the ICD-10-GM (International Classification of Diseases) Version 2018 [31].

### Preoperative evaluation and surgical approach

In each patient with suspected or histologically confirmed HCC an accurate tumor staging was performed including preoperative helical computed tomography (CT) scanning of the chest, abdomen, and pelvis and if necessary additional magnetic resonance imaging (MRI) scans of the liver to evaluate number of nodules, size and location of the tumor. Patients with preoperative hepatic disease were referred to expert hepatologists to assess and optimize underlying hepatic malfunction if feasible. After complete tumor staging all HCC patients were discussed in a multidisciplinary oncological board. General treatment consensus was based on multiple factors such as age, general health and performance status, co-morbidities and extent of liver disease. Distant metastases or lymph node involvement per se were not exclusion criteria for surgical resection. In all cases, an individual therapeutic approach guided by a rigorous risk-versus-benefit assessment was intended. In patients designated for surgical resection with prospective FLRV of < 30% and/or hepatobiliary scintigraphy results below the cut-off of 2.69%/min/m^2^ [32] hepatic augmentation techniques including either portal venous embolization (PVE) or in situ split plus portal vein ligation (ISLT) were applied. All included patients underwent open surgical resection. After careful initial surgical exploration intraoperative hepatic ultrasound evaluation was routinely conducted to assess tumor extent and resectability, and to rule out undetected tumor nodules. Parenchyma dissection was performed using the cavitron ultrasonic surgical aspirator (CUSA®; Valleylab, Boulder, Colorado, USA) without intermittent Pringle’s maneuver. Our technical approach of parenchymal in situ splitting has been recently described [33]. All the procedures were performed by the involved attending staff surgeons at the time of scheduled surgery. Of note, in patients undergoing ISLT a weekly volumetric and scintigraphic evaluation was performed to reassure adequate volume and function gain prior to the completion procedure.

### Follow-up

After hospital discharge each patient remained attached to our outpatient clinic or the referral oncologist and general practitioner for follow-up assessment. The follow-up routine consisted of physical examination and tumor marker evaluation, as well as ultrasonography and computed tomography or magnetic resonance imaging every 3 months within the first year after surgery extended to every 6 months beginning from the second postoperative year. Tumor recurrence was defined as the radiological or histological confirmation of newly detected tumor lesions after hepatic resection. Consecutively, DFS was defined as the time interval between hepatic resection and the onset of a new recurrence or last follow-up date while OS was defined as the time span from surgical resection to the occurrence of death from any cause respectively last follow-up.

### Statistical analysis

Data derived from continuous variables were expressed as median ± standard deviation (SD) and assessed using either the t-test or the Mann–Whitney U test. Categorical data were summarized as frequencies (%) and were compared using Fisher’s exact or chi-square test. Survival curves were created with the Kaplan–Meier method and compared by the log-rank test. Univariate and multivariate Cox regression analyses were conducted to identify predictive survival factors. All relevant clinical and pathological variables with *p* ≤ 0.1 in the univariate analysis were entered into the regression analysis using the forward stepwise variable selection. Hazard ratios (HRs) with 95% confidence intervals (CIs) were estimated. The OS analysis was not tumor associated.

All analysis were performed using missing values imputation appraising bias that arises from missing clinical data in the patient cohort. Variables with missing values > 20% were excluded from the analysis. Statistical analysis was performed using SPSS 25.0 software program (Statistical Package for Social Sciences; SPSS Inc., Chicago, IL, USA). A *p*-value < 0.05 was considered significant.

## Results

### Clinical characteristics and histopathological results

A total of 188 HCC patients (142 male /46 female) underwent curative resection at our institution between 2004 and 2021 and were eligible for the final analysis. Patient characteristics and the histopathological results are summarized in Table 1. The median age of the entire cohort was 68.50 ± 10.96 years (range 19–84 years) and the elderly population aged ≥ 70 years consisted of 89 (47.34%) patients. The median BMI of the entire study cohort was 25.97 ± 4.38. The majority of patients belonged to the ASA class III/V group (*n* = 124; 65.96%). Underlying hepatitis A, B and C infection was evident in 20 (10.64%), 42 (22.34%), and 56 (29.79%) patients respectively while alcoholic liver disease was documented in 37 (19.68%) patients. The liver function status based on the Child–Pugh classification was Child-A in 143 patients (76.06%), Child-B in 22 patients (11.70%) and Child-C in one patient (0.53%), whereas 22 patients (11.70) demonstrated no significant preoperative signs of liver cirrhosis. The median MELD score of all patients was 8.00 ± 3.52. Cardio-pulmonary disease were the most common co-morbid condition with 57.44%, followed by diabetes mellitus (34.04%), and chronic renal insufficiency (14.36%). Multiple HCC lesions were detected in 77 (40.96%) patients. The tumors of 134 (71.28%) patients were limited to one liver lobe. Only 5 (2.66%) patients had metastatic tumor spread. Based on preoperative imaging the median tumor diameter was 45.0 ± 48.48 mm. After final histopathological examination the tumors were classified as T-stage I/II and Grade I/II in 149 (79.26%) and 157 (83.51%) patients respectively. Vascular tumor infiltration was noted in 32 (17.02%) patients. The rate of R > 0.1 mm tumor clearance was 82.45%. Tumor infiltration and metastases into adjacent non-hepatic tissue were observed in 5 patients (2.66%). According to the UICC staging classification, 111 (59.04%) patients were stratified in stage I, 43 (22.87%) in stage II, 28 (14.89%) in stage III and 6 (3.19%) in stage IV A/B.

**Table 1:** Patient-histopathological characteristics and perioperative course

Variables	All patients (*n* = 188)
Age (years), [median ± SD]	68.50 ± 10.96
Age ≥ 70 years (n; %)	89 (47.34)
*Sex (n;%)*	
Male/Female	142/46 (75.53/24.47)
BMI (kg/m^2^), [median ± SD]	25.97 ± 4.38
ASA Score (n; %)	
ASA I/II	64 (34.04)
ASA III/IV	124 (65.96)
Hepatitis A (n; %)	20 (10.64)
Hepatitis B (n; %)	42 (22.34)
Hepatitis C (n; %)	56 (29.79)
CHILD–Pugh Score (n; %)	
No cirrhosis	22 (11.70)
A	143 (76.06)
B	22 (11.70)
C	1 (0.53)
Alcohol abuse (n; %)	37 (19.68)
MELD Score (median ± SD)	8.00 ± 3.52
***Co-morbidities (n; %)***	
Cardiac	65 (34.57)
Pulmonary	43 (22.87)
Renal	27 (14.36)
Diabetes mellitus	64 (34.04)
Tumor diameter (mm), [median ± SD]	45.00 ± 48.48
Single lesion (n; %)	111 (59.04)
Multiple lesions (n; %)	77 (40.96)
Unilobular lesion(s) (n; %)	134 (71.28)
Bilobular lesions (n; %)	54 (28.72)
***Pathology (n;%)***	
T-stage	
I/II	149 (79.26)
III/IV	39 (20.74)
M-stage	
M0	183 (97.34)
M1	5 (2.66)
Grade	
I/II	157 (83.51)
III/IV	31 (16.49)
L-Stage	
L0	178 (94.68)
L1	10 (5.32)
V-Stage	
V0	156 (82.98)
V1 (micro)	29 (15.43)
V2 (macro)	3 (1.60)
UICC-Stage	
I	111 (59.04)
II	43 (22.87)
III	28 (14.89)
IV	6 (3.19)
Resection margin	
R < 0.1 cm	33 (17.55)
R ≥ 0.1 cm	155 (82.45)
R < 0.5 cm	72 (38.30)
R ≥ 0.5 cm	116 (61.70)
***Operative data***	
ISLT/PVE (n; %)	15 (7.98)
Resected segments (n), [median ± SD]	2.00 ± 1.37
Segments ≥ 3 (n; %)	83 (44.15)
Biliary reconstruction (n; %)	14 (7.45)
T-Drain (n; %)	38 (20.21)
Intraoperative transfusion (n; %)	74 (39.36)
Operative time (min), [median ± SD]	307.00 ± 135.04
Blood units (BU), [median ± SD]	0.00 ± 9.69
***Postoperative outcome (n; %)***	
Bile leakage	20 (10.64)
Intra-abdominal abscess	14 (7.45)
Cholangitis	13 (6.91)
ISGLS B/C	58 (30.85)
Wound infection	24 (12.77)
Sepsis	19 (10.11)
Clavien-Dindo ≥ 3a	73 (38.83)

*ASA* American Society of Anesthesiologists, *BMI* body mass index, *ISGLS* International Study Group of Liver Surgery, *ISLT/PVE* in situ split plus portal vein ligation/portal venous embolization, *MELD* Model of End Stage Liver Disease, *UICC* Union internationale contre le cancer

### Operative data and short-term postoperative outcome

The rate of major hepatectomy (≥ 3 segments) was 44.15% with a median of 2.00 ± 1.37 resected segments in the entire cohort. In 15 (7.98%) patients with locally advanced HCC, augmentation techniques (PVE and/or ISLT) were necessary to increase the FLR. Complex biliary reconstruction was conducted in 14 (7.45%) patients and the rate of biliary T-tube insertion was 20.21%. The median operative time was 307.00 ± 135.04 min. During the operative procedure 74 (39.36%) patients received blood transfusions. Major postoperative morbidity (CD ≥ 3a) occurred in 73 (38.83%) patients. Among the postoperative complications, advanced liver failure ISGLS B/C was noticed most frequently (*n* = 58; 30.85%). Other morbidities included wound infection (*n* = 24; 12.77%), bile leakage (*n* = 20; 10.64%), sepsis (*n* = 19; 10.11%), intra-abdominal abscess (*n* = 14; 7.45%), and cholangitis (*n* = 13; 6.91%). Table 1 displays the intra-and postoperative course in detail.

### Overall and disease-free survival analysis

**Fig. 1:**
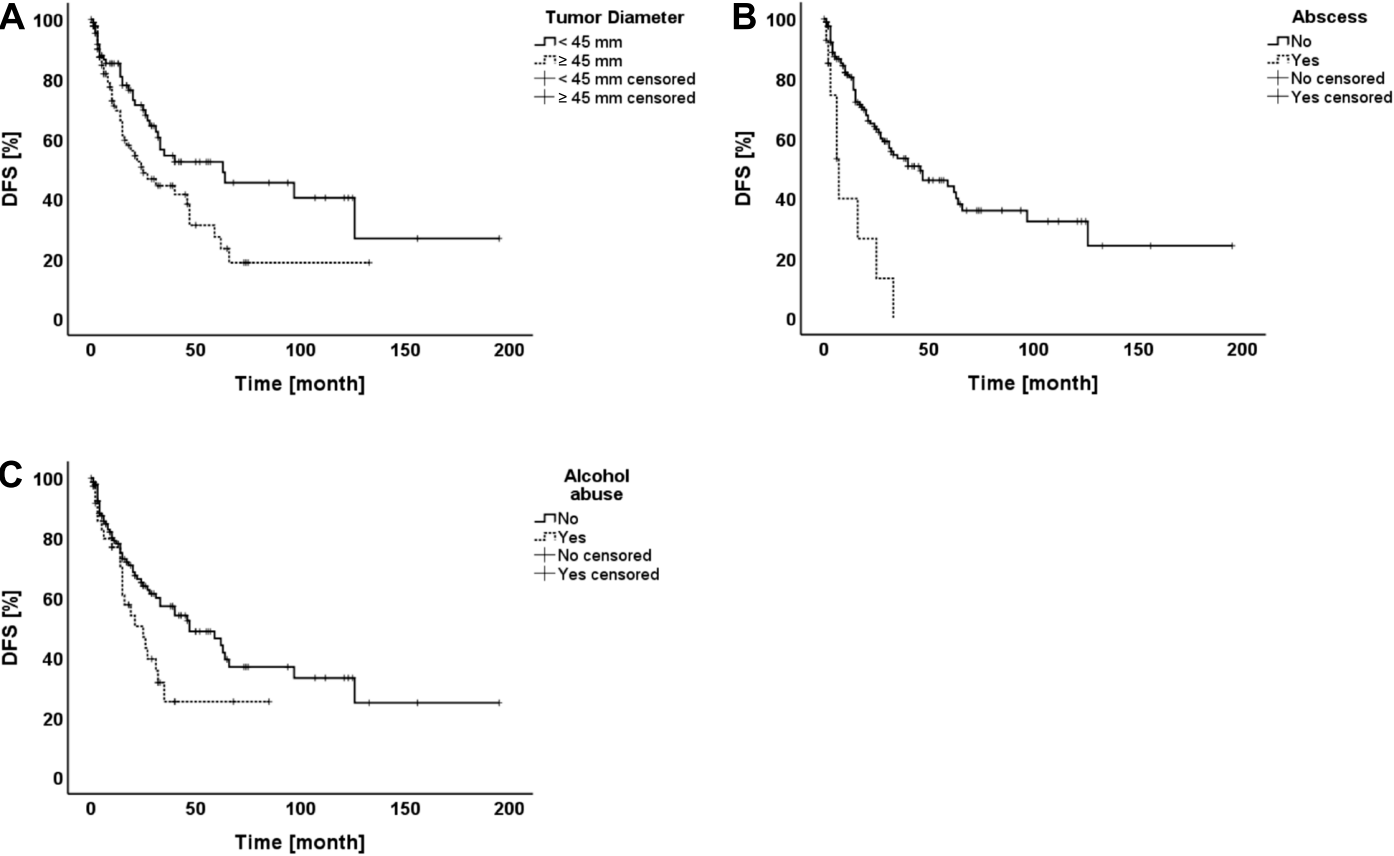
DFS curves according to significant predictive factors: **a** Tumor Diameter **b** Intra-abdominal abscess **c** Alcohol abuse

**Fig. 2:**
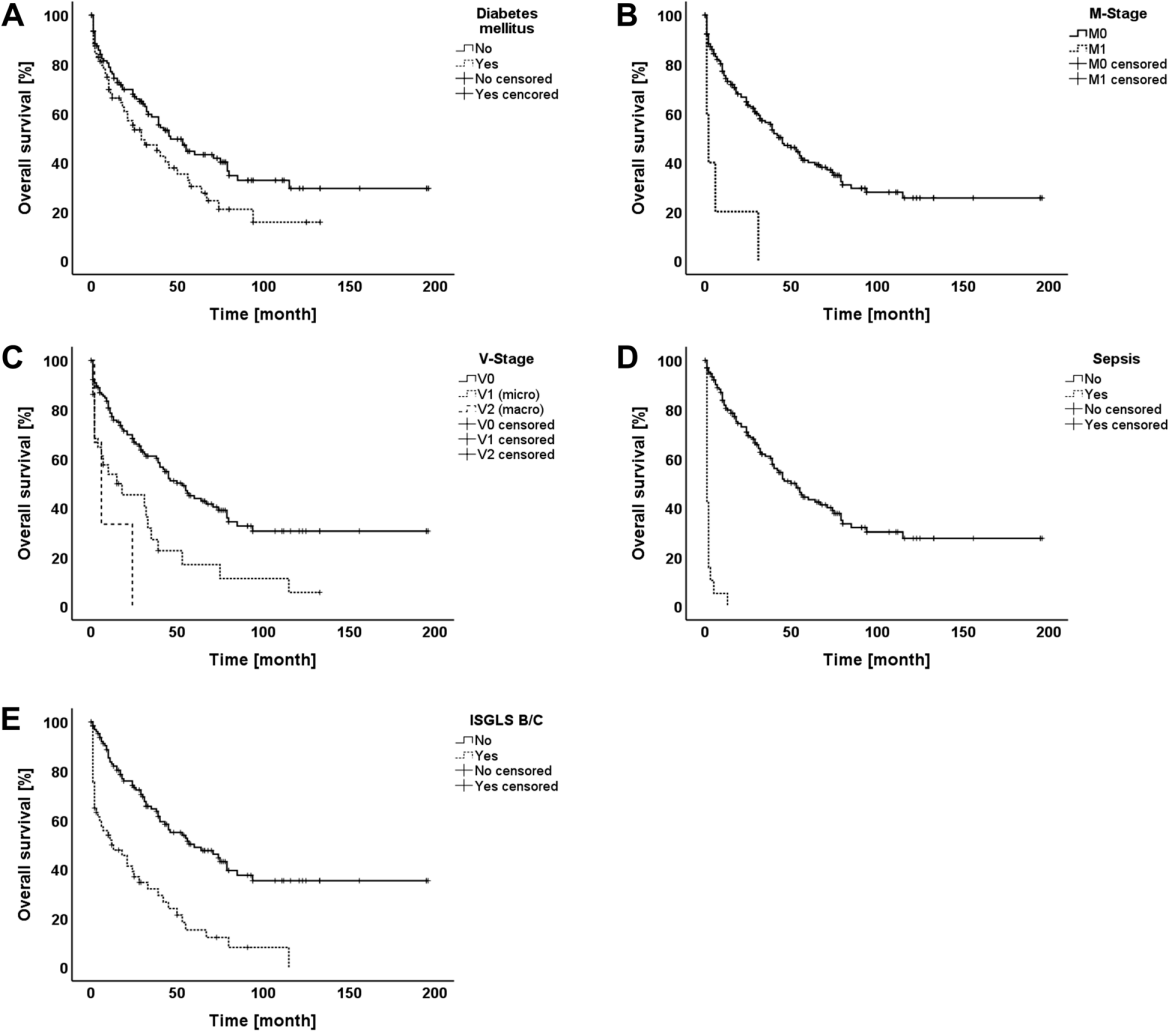
OS curves according to significant predictive factors: **a** Diabetes mellitus **b** M-Stage **c** V-Stage **d** Sepsis **e** ISGLS B/C

After a median follow-up of 27 months (range 1–196 months), 78 of 188 patients (41.49%) experienced disease recurrence. The site of relapse was intrahepatic in 71.79%, extrahepatic in 5.13% and synchronous intra-and extrahepatic tumor recurrence was noted in 23.08%. The actual 1, 3, and 5 year DFS rates were 77.9%, 49.7%, and 41% respectively. A total of 108 patients (57.45%) died within the follow-up period. The causes of death in these cases were HCC recurrence (*n* = 32; 29.63%), liver or multi-organ failure (*n* = 41; 37.96%), and other causes (*n* = 35; 32.41%). The 1, 3, and 5 year OS rates were 72.7%, 54.7%, and 38.8% respectively. Table 2 outlines the univariate analysis of predictive variables for DFS and OS. Accordingly, univariate analysis revealed that Hepatitis A (*p* = 0.064), chronic alcohol abuse (*p* = 0.027), tumor diameter ≥ 45 mm (*p* = 0.019), M-Stage (*p* = 0.003), UICC-Stage (*p* = 0.006), and intra-abdominal abscess (*p* < 0.0001) were significant risk factors for DFS whereas the following variables were associated with OS: Age ≥ 70 years (*p* = 0.027), BMI ≥ 25.97 kg/m^2^ (*p* = 0.054), Child–Pugh Score (*p* = 0.018), diabetes mellitus (*p* = 0.040), tumor diameter ≥ 45 mm(*p* = 0.005), T-Stage (*p* = 0.033), M-Stage (*p* < 0.0001), tumor grade (*p* = 0.019), L-Stage (*p* = 0.003), V-Stage (*p* < 0.0001), UICC-Stage (*p* < 0.0001), ISLT/PVE (*p* = 0.049), resected segments ≥ 3 (*p* = 0.002), biliary reconstruction (*p* = 0.003), CD ≥ 3a (*p* < 0.0001), intra-abdominal abscess (*p* = 0.051), cholangitis (*p* < 0.0001), sepsis (*p* < 0.0001), and ISGLS B/C (*p* < 0.0001). The significant factors identified by univariate analysis for DFS and OS were consecutively included into a multivariate Cox regression model. Tables 3 and 4 show the results of the multivariate analysis. Hence, tumor diameter ≥ 45 mm [HR 1.725; (95% CI 1.091–2.727); *p* = 0.020], intra-abdominal abscess [HR 3.812; (95% CI 1.859–7.815); *p* < 0.0001], and preoperative chronic alcohol abuse [HR 1.831; (95% CI 1.102–3.042); *p* = 0.020] were independently predictive for DFS (Fig. 1a-c). On the other hand, diabetes mellitus [HR 1.714; (95% CI 1.147–2.561); *p* = 0.009), M-Stage [HR 2.656; (95% CI 1.034–6.826); *p* = 0.042], V-Stage [HR 1.946; (95% CI 1.299–2.915); *p* = 0.001, Sepsis [HR 10.999; (95% CI 5.167–23.412); *p* < 0.0001], and ISGLS B/C [HR 2.008; (95% CI 1.273–3.168); *p* = 0.003] were independently associated with OS (Fig. 2a-e).

**Table 2:** Univariate analysis of predictive variables for DFS and OS

Variables	Number (n;%)	DFS HR (95% CI)	*P*-value	OS HR (95% CI)	*P*-value
Age		1.018 (0.646–1.603)	0.939	1.522 (1.040–2.227)	0.027
≥ 70 years	89 (47.34)				
< 70 years	99 (52.66)				
Sex		0.831 (0.503–1.373)	0.464	1.031 (0.654–1.627)	0.894
Male	142(75.53)				
Female	46 (24.47)				
BMI		0.744 (0.475–1.165)	0.190	0.693 (0.473–1.013)	0.054
≥ 25.97 kg/m^2^	92 (48.93)				
< 25.97 kg/m^2^	96 (51.07)				
ASA Score		0.755 (0.480–1.187)	0.217	1.271 (0.846–1.909)	0.242
I/II	64 (34.04)				
III/IV	124(65.96)				
Hepatitis A		0.401 (0.146–1.100)	0.064	0.965 (0.517–1.801)	0.909
Yes	20 (10.64)				
No	168 (89.36)				
Hepatitis B		1.101 (0.661–1.833)	0.709	0.720 (0.442–1.170)	0.178
Yes	42 (22.34)				
No	146 (77.66)				
Hepatitis C		0.723 (0.426–1.226)	0.222	0.829 (0.536–1.281)	0.392
Yes	56 (29.79)				
No	132 (70.21)				
Child–Pugh Score		0.991 (0.628–1.563)	0.964	1.419 (0.966–2.084)	0.018
No cirrhosis	22 (11.70)				
A	143 (76.06)				
B	22 (11.70)				
C	1 (0.53)				
*Co-morbidity*					
Cardiac		0.666 (0.392–1.130)	0.126	1.310 (0.885–1.939)	0.170
Yes	65 (34.57)				
No	123 (65.43)				
Pulmonary		1.039 (0.612–1.763)	0.888	1.301 (0.853–1.984)	0.214
Yes	43 (22.87)				
No	145 (77.13)				
Renal insufficiency		0.740 (0.356–1.541)	0.415	1.159 (0.681–1.973)	0.580
Yes	27 (14.36)				
No	161 (85.64)				
Diabetes mellitus		1.402 (0.887–2.215)	0.142	1.491 (1.010–2.200)	0.040
Yes	64 (34.04)				
No	124 (65.6)				
Chronic alcohol abuse		1.736 (1.052–2.866)	0.027	1.121 (0.716–1.754)	0.614
Yes	37 (19.68)				
No	151 (80.32)				
MELD Score		1.323 (0.842–2.078)	0.218	1.360 (0.926–1.999)	0.111
≥ 8	107 (56.91)				
< 8	81 (43.09)				
Tumor Diameter		1.697 (1.081–2.666)	0.019	1.715 (1.164–2.526)	0.005
≥ 45 mm	97 (51.59)				
< 45 mm	91 (48.41)				
Single lesion	111 (59.04)	1.259 (0.806–1.968)	0.306	1.284 (0.880–1.873)	0.188
Multiple lesions	77 (40.96				
Unilobular lesion(s)	134 (71.28)	1.455 (0.898–2.355)	0.121	1.292 (0.863–1.935)	0.206
Bilobular lesions	54 (28.72)				
T-Stage		1.129 (0.642–1.985)	0.669	1.583 (1.029–2.436)	0.033
I/II	149 (79.26)				
III/IV	39 (20.74)				
M-Stage		6.672(1.554–28.642)	0.003	5.106 (2.043–12.760)	< 0.0001
M0	183 (97.34)				
M1	5 (2.66)				
Grade		1.284 (0.677–2.438)	0.438	1.773 (1.085–2.896)	0.019
I/II	157 (83.51)				
III/IV	31 (16.49)				
Resection margin					
R < 0.1 cm	33 (17.55)	1.216 (0.701–2.107)	0.482	1.174 (0.729–1.892)	0.504
R > 0.1 cm	155 (82.45)				
R < 0.5 cm	72 (38.30)	1.105 (0.698–1.751)	0.667	0.906 (0.617–1.330)	0.610
R > 0.5 cm	116 (61.70)				
L-Stage		1.708 (0.623–4.683)	0.288	2.833 (1.370–5.859)	0.003
L0	178 (94.68)				
L1	10 (5.32)				
V-Stage		0.811 (0.406–1.622)	0.594	2.249 (1.539–3.287)	< 0.0001
V0	156 (82.98)				
V1 (micro)	29 (15.43)				
V2 (macro)	3 (1.60)				
UICC-Stage		1.267 (0.962–1.669)	0.006	1.512 (1.227–1.863)	< 0.0001
I	111 (59.04)				
II	43 (22.87)				
III	28 (14.89)				
IV	6 (3.19)				
ISLT/PVE		1.393 (0.561–3.454)	0.468	1.839 (0.984–3.434)	0.049
Yes	15 (7.98)				
No	173 (92.02)				
Segments ≥ 3		1.171 (0.745–1.842)	0.489	1.783 (1.221–2.606)	0.002
Yes	83 (44.15)				
No	105 (55.85)				
Biliary reconstruction		1.162 (0.364–3.707)	0.797	2.417 (1.323–4.416)	0.003
Yes	14 (7.45)				
No	174 (92.55)				
T-Drainage		1.456 (0.874–2.425)	0.143	0.902 (0.560–1.453)	0.667
Yes	38 (20.21)				
No	150 (79.79)				
Operative time		0.710 (0.451–1.118)	0.134	1.356 (0.927–1.984)	0.111
≥ 307 min	94 (50.00)				
< 307 min	94 (50.00)				
Intraoperative transfusion		0.708 (0.437–1.147)	0.155	1.248 (0.852–1.828)	0.248
Yes	74 (39.36)				
No	114 (60.64)				
CD ≥ 3a		1.351 (0.845–2.160)	0.202	2.272 (1.555–3.319)	< 0.0001
Yes	73 (38.83)				
No	115 (61.17)				
Bile leak		1.168 (0.600–2.276)	0.644	0.966 (0.529–1.761)	0.908
Yes	20 (10.64)				
No	168 (89.36)				
Intra-abdominal abscess		4.152 (2.036–8.467)	< 0.0001	1.755 (0.982–3.137)	0.051
Yes	14 (7.45)				
No	174 (92.55)				
Cholangitis		0.829 (0.203–3.386)	0.792	2.885 (1.541–5.402)	< 0.0001
Yes	13 (6.91)				
No	175 (93.09)				
Sepsis		1.218 (0.161–9.221)	0.847	19.225 (9.947–37.159)	< 0.0001
Yes	19 (10.11)				
No	169 (89.89)				
Wound infection		0.948 (0.486–1.846)	0.873	1.332 (0.802–2.210)	0.260
Yes	24 (12.77)				
No	164 (87.23)				
ISGLS B/C		1.287 (0.755–2.193)	0.347	2.992 (2.026–4.420)	< 0.0001
Yes	58 (30.85)				
No	130 (69.15)				

*ASA* American Society of Anesthesiologists, *BMI* body mass index, *CD* Clavien-Dindo, *HR* hazard ratios, *ISGLS* International Study Group of Liver Surgery, *ISLT/PVE* in situ split plus portal vein ligation/portal venous embolization, *MELD* Model of End Stage Liver Disease, *UICC* Union internationale contre le cancer

**Table 3:** Multivariate analysis of predictive OS factors

Multivariate Analysis		
**Variables**	**HR (95% CI)**	***P*-Value**
Diabetes mellitus	1.714 (1.147–2.561)	0.009
M-Stage	2.656 (1.034–6.826)	0.042
V-Stage	1.946 (1.299–2.915)	0.001
Sepsis	10.999 (5.167–23.412)	< 0.0001
ISGLS B/C	2.008 (1.273–3.168)	0.003

**Table 4:** Multivariate analysis of predictive DFS factors

Multivariate Analysis		
**Variables**	**HR (95% CI)**	***P*-Value**
Tumor Diameter ≥ 45 mm	1.725 (1.091–2.727)	0.020
Intra-abdominal abscess	3.812 (1.859–7.815)	< 0.0001
Preoperative chronic alcohol abuse	1.831 (1.102–3.042)	0.020

## Discussion

Tumor recurrence following curative hepatectomy for HCC is a commonly observed problem accounting for poor survival rates [21]. In the presented study, we identified several factors which inversely affect recurrence-free and overall survival in a western cohort of mainly cirrhotic patients undergoing HR. Multivariate cox regression analysis revealed that tumor diameter ≥ 45 mm, intra-abdominal abscess formation, and preoperative chronic alcohol abuse are significant predictive parameters of tumor recurrence. OS is independently influenced by diabetes mellitus, M-Stage, V-Stage, sepsis, and postoperative liver failure ISGLS B/C. In our patient cohort, the overall 1-, 3- and 5 year DFS rates were 77.9%, 49.7%, and 41% respectively. The corresponding 1-, 3- and 5 year OS rates were 72.7%, 54.7%, and 38.8% respectively. However, different study populations with various liver conditions may influence the generalization of our findings, as our short-and midterm outcome data differ from previously reported European and Asian results [34–36].

Postoperative HCC recurrence is considered to be closely related to patho-biological tumor features [37, 38]. Here, tumor size has been shown to be an accurate and independent predictor of HCC response [39]. Indeed, larger tumor diameter is directly associated with early recurrence after surgical resection and transplantation [40, 41]. The aneuploid DNA content in HCC tumors exceeding 3 cm leads to a highly aggressive behavior and thus to a worse survival [42]. In the literature, a wide range of prognostic tumor size cut-off values from 2.6–10 cm have been previously suggested [43, 44]. Our analysis revealed a tumor size ≥ 4.5 cm as a significant parameter of recurrence. Tumor diameter is consequently a key component of clinical prognostic tools such as the Milan criteria [45] and the University of California, San Francisco criteria [46]. Beside tumor size, the AFP (alpha-fetoprotein) score is widely applied to assess postoperative prognosis as it correlates with vascular invasion and differentiation in HCC [47, 48]. Recently Mazzotta et al. [49] have shown that, in addition to the AFP score, the number of HCC lesions (≥ 5 nodules) before transplantation has a significant impact on overall survival. Therefore, the combination of AFP score and tumor number is recommended to exclude high-risk listed patients and to accurately predict oncological outcome after liver transplantation [49].

Vascular tumor involvement either as macroscopic or microscopic vessel invasion represents another pivotal characteristic related to high tumor recurrence and disadvantageous outcome [50, 51]. It has been demonstrated that macro-vascular invasion is associated with an approximately fourfold decrease in time-to-recurrence and reduced long term survival [52, 53]. Micro-vascular invasion (MVI) is defined as the presence of tumor emboli within the central hepatic vein, the portal vein, or the large capsular vessels [54]. Several studies identified MVI as an independent factor for early recurrence [19, 55, 56]. However, despite discouraging prognosis in patients with vascular tumor involvement, hepatic resection still achieves superior 1-year survival rates compared to other treatment options and best supportive care [53]. In the presented analysis, vascular infiltration was an independent predictive factor of OS but not DFS. We therefore speculate that vascular tumor involvement results in a higher rate of extended hepatectomies to achieve clear margins, which in turn could be associated with major complications and decreased survival within 90 postoperative days. Thirty-two patients (17.02%) of our cohort showed vascular invasion [microscopic *n* = 29; (15.43%), macroscopic *n* = 3; (1.60%)] and 20 major hepatic resections were performed in this subgroups accounting for a 35% 90-day mortality rate. In various cancer types, distant metastases as an expression of advanced tumor burden were associated with a rather unfavorable survival. A large data-based study [57] analyzed the outcome of patients with locally resectable HCC and extrahepatic metastasis and compared the results with a cohort of not-resected patients. Hepatic resection had a favorable impact on the prognosis of HCC in this specific subgroup of patients. In contrast, at the time of surgery distant metastases were present in 5 patients of our study cohort and M-Stage was independently associated with a poor OS.

It is already known that diabetes mellitus (DM) triggers hepatic carcinogenesis through various pathophysiological mechanisms [58, 59] and there is epidemiological evidence suggesting that diabetes increases the risk of HCC prevalence and mortality [60]. However, inconsistent findings were reported regarding the prognostic impact of diabetes mellitus in patients with HCC undergoing curative resection [61, 62]. A previously conducted meta-analysis with 16 included studies demonstrated that DM was associated with an increased risk of overall postoperative complications and unfavorable DFS and OS after hepatectomy [63]. Our findings are in-line with the results from Wang et al. [61] highlighting the negative influence of DM on OS in cirrhotic HCC patients. Alcohol consumption also increases the risk of HCC mainly due to the development of liver cirrhosis. The prevalence of alcohol-induced HCC is higher in Western countries compared to the Asian territory where HCC is predominately related to viral infection [64, 65]. Preoperative alcoholic intake was identified as an independent risk factor of poor DFS in HCC patients after hepatectomy. The severity of alcohol consumption has significantly influenced DFS rates [66]. Furthermore, resumption of abusive alcohol drinking after LT has been shown to correlate with poor long-term survival [67]. Of note, our observation is based on evaluation of preoperative alcoholism, whereas persistent alcohol consumption was not documented during the follow-up examinations.

The presence of postoperative complications plays an important role in the prospective disease course of HCC patients following HR. Patients with postoperative complications showed a significant reduction in OS [HR 1.39; 95% CI (1.28–1.50); *p* < 0.0001] and a worse DFS [HR 1.25; 95% (CI 1.16–1.35); *p* < 0.001] in comparison to patients without postoperative morbidity irrespective of the complication severity as demonstrated in a meta-analysis with 14.096 included patients [68]. Among postoperative complications, intra-abdominal infection and sepsis were found to be significant predictors of both poor recurrence-free and overall survival in liver resection for HCC and colorectal liver metastases respectively [69, 70]. A possible explanation relies in the fact that postoperative infectious complications may be involved in an excessive and sustained systemic pro-inflammatory response promoting adhesion and the invasive capacity of circulating cancer cells alongside functional impairment of anti-tumor immune cells including cytotoxic T cells and natural killer cells [71–73].

The ISGLS criteria of posthepatectomy liver failure (PHLF) have reliably stratified patients according to the risk of early postoperative mortality following HR [74, 75]. Recently, Fukushima et al. [76] addressed the value of ISGLS liver failure criteria in patients with HCC resection demonstrating its significant impact on both DFS and OS. In contrast, our study was only able to find a correlation between ISGLS criteria Grade B/C and OS.

Liver cirrhosis per se has been identified as a risk factor of late recurrence regarding de novo HCC formation when compared to patients with viral hepatitis without cirrhosis [50, 54]. The majority of our included patients (*n* = 166; 88.29%) had different degrees of liver cirrhosis. Interestingly, the 3-and 5 year DFS rates in our study were markedly higher in comparison to Penzkofer et al. (3-and 5 year DFS of 20%, and 7% respectively) who analyzed survival in non-cirrhotic HCC patients after resection [34]. Another recent study [77] identified that DFS and OS rates were significantly influenced by perioperative blood product transfusions in HCC but our study results did not show this correlation.

The wide range of clinical implication of these elucidated predictive parameters are obvious. Survival benefit in patients with HR based on pre-operative variables raises the question which patients might oncologically benefit most from surgery and which individual factors could be optimized prior resection. Moreover, as demonstrated, postoperative morbidity plays an important role in both DFS and OS. Therefore, efforts should be made to minimize these complications or to effectively treat them. Taken together, predictive survival factors should be incorporated into the patient tailored treatment and postoperative surveillance strategy.

However, our study has some important limitations given the retrospective protocol with a relatively small sample size. Furthermore, data were derived from a single European institution without a possible validation in a large multi-center control cohort. It is noteworthy that the treatment of tumor recurrence and its effects on survival were not evaluated. In deceased patients it was not always possible to strictly discriminate between sepsis and single/or multiorgan failure as the leading cause of death. Importantly, the analysis is subject to a selection and missing values bias as patient allocation and operative strategy were determined by the institutional approach and preference of the involved surgeons and cases with missing or incomplete follow-up data were excluded from the analysis. In view of the long study interval, advances in surgical and non-surgical treatment practice, improved perioperative management and, above all, better tumor follow-up programs must be taken into account when interpreting the results.

## Conclusions

Various oncological and non-oncological parameters were identified predicting DFS and OS in patients undergoing curative HR for HCC. The implementation of these factors into interdisciplinary treatment concepts and adjustment of perioperative modifiable non-oncological variables could further improve oncological outcome. Larger scaled and multi-institutional studies with comparable patient cohorts are needed to further validate the presented results.

## Data Availability

The data presented in this study are available upon request from the corresponding author.

## Abbreviations

HCC: Hepatocellular carcinoma
DFS: Disease-free survival
OS: Overall survival
HR: Hepatic resection
LT: Liver transplantation
STROBE: Strengthening the Reporting of Observational Studies in Epidemiology checklist
BMI: Body mass index
ASA: American Society of Anesthesiologists
MELD: Model for End-Stage Liver Disease
HBS: Hepatobiliary scintigraphy
FLR: Future liver remnant
ISGLS: International Study Group of Liver Surgery
UICC: Union internationale contre le cancer
CD: Clavien-Dindo
ICD: International Classification of Diseases
CT: Computed tomography
MRI: Magnetic resonance imaging
PVE: Portal venous embolization
ISLT: In situ split plus portal vein ligation
CUSA: Cavitron ultrasonic surgical aspirator
SD: Standard deviation
HRs: Hazard ratios
CIs: Confidence intervals
AFP score: Alpha-fetoprotein
MVI: Micro-vascular invasion
DM: Diabetes mellitus
PHLF: Posthepatectomy liver failure
